# Mobilising strategic alliances with community organisations to address work-related mental injury: a qualitative study guided by collaboration theory

**DOI:** 10.1186/s12889-023-17170-w

**Published:** 2023-11-16

**Authors:** Corina Crisan, Pieter Andrew Van Dijk, Jennifer Oxley, Andrea De Silva

**Affiliations:** 1 Monash Sustainable Development Institute, Monash University, Melbourne, Australia; 2 Monash Business School, Monash University, Melbourne, Australia; 3 Monash University Accident Research Centre, Monash University, Melbourne, Australia; 4 School of Public Health and Preventive Medicine, Monash University, Melbourne, Australia

**Keywords:** Collaboration theory, Work-related mental injury, Prevention, Help-seeking, Community organisations, Strategic alliances, Framework development, Australia

## Abstract

**Background:**

A critical policy issue in Australia and worldwide is the escalating rates of work-related mental injury that have been linked to the lack of help-seeking behaviours of at-risk workers. Strategic alliances between community organisations, statutory bodies, and mental health service providers could expand the efficacy and reach of mental health literacy and peer support initiatives that can encourage help-seeking, however, there is limited evidence to support the development of such approaches. This study used a qualitative design based on collaboration theory to explore the factors influencing community organisation leaders’ decisions to provide such initiatives through collaboration with relevant third parties.

**Methods:**

Repositories of submissions into mental health reviews and publicly available registers in Australia were used to identify twenty-two participant organisations (n = 22), which were categorised according to the International Classification of Non-Profit Organisations (Culture & Recreation, Social Services, and Development & Housing). Eleven of these organisations demonstrated an interest in collaborating with third parties and extending efforts to deliver work-related mental health initiatives through contributions to mental health reviews. Leaders were interviewed to understand differences in perspectives on potential collaborations.

**Results:**

Organisations that did not make submissions were reluctant to engage in such efforts due to limitations in expertise/capacity, and perceived mission misalignment. Third-party support from statutory bodies and mental health service providers addressing these perceived limitations may improve their confidence, and willingness to engage. Regardless of their category, all considered the benefit of such collaboration included improving the acceptability, approachability, availability, and efficacy of work-related mental health initiatives. Equity was seen as supporting decision-making/leadership, while power imbalance was a barrier. Third-party contributions that could facilitate collaboration included expert support/credibility, administration, formal structures, supportive policy, and joining networks, however, red tape was a challenge. Shared values, vision, practice, and networking were identified as supporting positive communication and interpersonal relations.

**Conclusion:**

The study establishes that, adequately supported and resourced, community organisations are willing to align strategically with statutory bodies and mental health service providers to use their unique position in the community to deliver work-related mental health literacy and peer support programmes for at-risk workers to improve help-seeking behaviours.

**Supplementary Information:**

The online version contains supplementary material available at 10.1186/s12889-023-17170-w.

## Highlights

This is the first study that used a qualitative approach guided by collaboration theory to explore the motivations influencing Community Organisation (CO) leaders’ decisions to work with statutory bodies and mental health service providers and deliver programmes aimed to improve workers’ access to mental health literacy resources and peer support.

Understanding the beliefs that differentiate COs that provided submissions into mental health reviews from those that did not is imperative in the design of effective strategies to facilitate future engagement.

The use of a theory-based decision-making model (the Strategic Alliances Framework) was critical in determining the scale, and types of initiatives that different categories of COs may be able to provide, and potential levels of engagement with relevant third parties.

COs must be adequately guided, resourced, and supported by statutory bodies/regulators and organisations with mental health expertise if they are to meet the contemporary needs of their communities and achieve impact beyond work-related mental health awareness raising.

**Supplementary Information:**

The online version contains supplementary material available at 10.1186/s12889-023-17170-w.

## Introduction

Work-related mental injury rates are escalating worldwide, and in Australia are estimated to cost $39 billion per year through productivity losses, worker turnover rates, compensation claims, and increased healthcare expenses [1]. The individual economic and social costs of these conditions for workers and their families are also considerable and are associated with reduced quality of life, social isolation, loss of income, and relationship breakdown [1]. Despite the provision of evidence-based programmes within the workplace and referral services that can build workers’ knowledge and skills to recognise, manage, and prevent mental illness [2], many of them are unwilling, or are unable to access them [3].

Addressing these increasing rates is a complex challenge for occupational health and safety (OHS) regulators/policymakers, and healthcare professionals in Australia [1] and worldwide [4]. A reflection of this complexity is that many workers fail to access timely professional or workplace assistance due to: a lack of awareness; stigma; belief that treatment is ineffective; fear of discrimination; unsupportive work cultures; concerns about negative job or career consequences; inaccessibility to suitable support; and/or a combination of these factors [3, 5]. The very cause and nature of work-related mental injury reflect some of the barriers to help-seeking within/through the workplace in that these conditions are linked to such things as job strain, harassment, bullying, violent or traumatic events, and interpersonal conflict [6, 7]. Adding to these barriers are fear of marginalisation and low mental health literacy [3, 8]. Compounding these problems is the isolation and insecurity of an increasing number of workers due to digitisation, and the contractual and global nature of modern work, which removes them from organisational support networks and resources [9]. Furthermore, there is increasing evidence of the effects of isolation on a large sector of the workforce during the COVID-19 pandemic [10]. Engaging this group of affected workers is a critical policy issue if we are to significantly reduce the long-term negative individual, societal, and productivity impacts of unaddressed work-related mental injury [11].

Previous research [12] and responses to recent inquiries into the Victorian [13] and broader Australian [1] mental health systems have identified community organisations (COs), as a potential complementary and effective asset to address some of the challenges in relation to the prevention and management of mental ill-health including work-related mental injury. Although the initiatives provided by COs are generic in nature, they could potentially be tailored to address work-related issues. Organisations such as sporting clubs and Men’s Sheds currently provide initiatives that raise awareness of mental illness [14] (i.e. RUOK?Day), use peers with lived experience to break down stigma and encourage help-seeking [15] and deliver education sessions to improve recognition of mental health conditions and provide support for those needing help [16] (i.e. Mental Health First Aid training). Importantly, the socially-inclusive environments, and relationship-based approaches promoted by COs are particularly useful in reaching marginalised individuals [17–19].

Recent evidence suggests that workers perceive COs as an appealing and safe option to source information and advice regarding the causes, nature, and treatment of work-related mental injury, as well as an opportunity to discuss personal experiences with peers [20]. However, COs are also seen as lacking the required expertise and skilled facilitators to address worker-specific mental health needs [20]. Alliances leveraging the authority, resources and infrastructure of statutory bodies, and the expertise of mental health service providers could build COs’ capacity to provide workers with opportunities to access mental health information and peer support. Though this may be the case, COs have not specifically been identified as potential partners partly due to a lack of qualification and resources to deliver initiatives beyond awareness-raising [16], but also because historically their missions do not align with this goal. However, a recent open call for suggestions to improve Australia’s mental health system (Productivity Commission mental health inquiry) [1] demonstrated that many COs are willing to direct efforts toward addressing work-related mental health.

While recent public inquiries, research, and the willingness of COs strengthen the case for aligning these organisations into OHS networks, there is little understanding of how strategic alliances can be achieved. Notwithstanding, there are broad theoretical approaches that can guide the establishment of sustainable collaborations to address work-related mental health. Collaboration theory includes general principles that can guide entities toward developing goals, and strategies to establish, and maintain collaborations between key partners [21, 22]. The Strategic Alliances Framework (SAF) [23], which is based on collaboration theory, provides a well-established framework that can be used to explore unique challenges and facilitators of building ongoing working relationships between COs, statutory bodies, and mental health service providers. This framework is particularly useful for policymakers and practitioners as it guides the identification of resources, processes, and structures that need to be in place to develop sustainable interorganisational relationships, having been used successfully to develop cross-sector collaborations in health and safety-related settings [23, 24]. More specifically, the framework helps identify the key factors that influence the development of working relationships among organisations into four domains, including *purpose* (the reason/s why organisations would join efforts and what they consider to be a common issue), *strategies and tasks* (how partners would coordinate their activities/operations to achieve the purpose of the alliance), *leadership and decision-making* (how the alliance should be led and decisions be made), and *communication and interpersonal relations* (the characteristics of relationships that would facilitate or challenge the productivity of the alliance). These factors are critical in determining whether potential partners/ships have, or can generate, the capacity, and characteristics to effectively respond to the target audience’s needs. Secondly, the model identifies various levels of strategic alliance along a continuum of integration including cooperation, coordination, and collaboration, and suggests a developmental path to move alliances on the continuum [23]. According to the literature, cooperation is best suited for parties aiming to disseminate information, coordination for those focussed on shaping programmes/policy, and collaboration for those willing to share resources/expertise and/or jointly deliver activities [25]. The model can also help determine where alliances with different COs may be placed on the continuum, based on their capacity and support required, and identify the factors that would help them become more integrated [23].

Therefore, guided by SAF [23], the overall aim of this study is to explore the factors influencing COs’ decisions to establish strategic alliances with key stakeholders and to what level, aimed to provide work-related mental health initiatives such as education and peer support. Previous research has used the United Nations International Classification of Non-Profit Organisations (ICNPO) [36] classification system and SAF to better understand the potential contribution of COs in addressing the mental health crisis in the UK [24, 26]. Our study looks to extend this research by determining the ICNPO categories of COs that may be suited to addressing work-related mental health at a community level in an Australian context. The SAF will be used to determine key facilitators and barriers for these organisations to work collaboratively with relevant statutory bodies and mental health service providers, in particular the levels of collaboration/integration with these entities in the design and delivery of work-related mental health initiatives.

This study contributes to the extant body of literature on health collaboratives involving COs [27–29]. It was designed to provide research-driven evidence for policymakers and practitioners to more fully understand the potential of currently under-utilised community resources in COs to help address escalating rates of work-related mental injury, particularly by looking to improve the help-seeking behaviours of at-risk workers. The results of this study can be used to inform the development of approaches that could guide Victorian statutory bodies and mental health service providers to develop a range of strategic alliances with COs that combine the resources, strengths, and expertise across three sectors to address a critical social and policy issue. The research questions that guided this research were:

RQ1 What categories of COs are suited to deliver work-related mental health information, education, and peer support programmes, and what would motivate them to do so?

RQ2 What is the capacity of these organisations to deliver work-related mental health information, education, and peer support programmes?

RQ3 What are the facilitators and barriers influencing COs’ decisions to work with relevant statutory bodies, and mental health service providers to tailor, and deliver work-related mental health information, education, and peer support programmes?

## Methods

COREQ guidelines for qualitative research were followed to ensure the transparency of reporting on research design and methods of data collection and analysis [30].

### Identifying, grouping and categorising participants

Two sets of data sources and previous research were used to categorise and select organisations for this study. The first were repositories of recent submissions (n = 3,256) to four federal, and one Victorian, Parliamentary inquiries into mental health systems [31–35]. These inquiries did not specifically target work-related mental health issues. The submissions required interested parties to demonstrate their capacity and interest to work with vested bodies, and direct organisational resources toward improving mental health and wellbeing outcomes in the community. Submissions were first screened to identify COs that expressed a willingness to collaborate and contribute to mental health reforms (n = 782). Submissions were further screened to determine if these organisations reflected the characteristics workers found appealing, which included that they served working-age groups, had formal legal structures, had reach in the community, were not mental health treatment providers, were not formally linked to workplace settings, and their avenues/missions fostered social participation [20]. At the conclusion of this process, we compiled a list of twenty-two COs (n = 22) and categorised them using the ICNPO [36] classification, to determine the types of activities/services they provided.

Next, we reviewed publicly available repositories such as the register of Australian charities and lists provided by voluntary organisations such as ‘Third Sector’ to identify COs that were also promoting mental health information and education initiatives within the community, although they did not provide submissions to the mental health reviews, and explore the conditions in which they would extend themselves as potential collaborators. Understanding the beliefs that differentiate COs that provided submissions from those that did not can help in the development of targeted strategies to facilitate future engagement. An additional twenty COs that possessed the attributes that workers found appealing in a mental health literacy programme provider [20] were identified (n = 20). Information regarding the purpose/mission, type of activities/services provided, size, legal structures, location, and characteristics of population groups serviced by these COs were extracted from their public records and recorded in Excel. At the conclusion of this process, we identified, grouped and categorised forty-two potential participant organisations (see Additional file 1: Appendix 1. Characteristics of Community Organisations identified and recruited).

### Recruitment

Ethics approval for the research was obtained from the Monash University Human Research Ethics Committee (project ID: 28867). Once potential participating organisations were identified, purposive sampling [37] was used to recruit organisational representatives based on their level of seniority (executive or senior managers) and tenure (> 12 months). Contact details of participants were sourced from their organisational submissions and/or websites. Leaders were contacted by email which included an explanatory statement, an invitation to participate in interviews and the contact details of the researchers if they chose to participate in the study. The information explained the purpose of the interviews was to explore the interest, capacity, and role that their organisation may have in delivering programmes that can educate workers about work-related mental health issues and encourage them to seek help, and explore the facilitators and barriers that would support or inhibit possible partnerships with statutory bodies and mental health service providers. The invitation established that no mental health assessment would be conducted, participation was anonymous and voluntary, and information collected would be confidential.

### Data collection

Data was collected using semi-structured interviews which were conducted via video platforms (Zoom/Microsoft Teams) over four months period between October 2021 and January 2022. Before commencing the interview, the purpose of the research was explained to each participant, informed consent was obtained, and demographic information was collected. Participants were informed that they could withdraw from the study at any time during the interview. The interviews were transcribed verbatim, excluding any identifying information. The length of interviews ranged from 28 to 52 minutes (average of 38 minutes). The final sample was considered by the research team to be representative and suitable for this study.

### Materials

Each participant was provided a description of work-related mental injury and examples of the types of mental health information, education, and peer support programmes (i.e., RUOK?Day and Mental Health First Aid training) that address these issues and could be provided by COs to promote help-seeking from appropriate sources such as workplace and public health services.

### Interview design

The interviews were conducted by the first author with postgraduate training in qualitative methods and facilitator experience. The interview schedule was piloted with four CO leaders from the research team’s professional network and subsequently refined prior to commencing data collection. These data were not included in the analysis. The interview schedule was structured in two sections. The first section contained open-ended questions designed to explore participants’ reasons for making or not making submissions to mental health reviews, and their views about the capacity of their organisation to address work-related mental health through providing mental health literacy programmes and/or peer support. The second section included questions aimed at exploring reasons COs would be interested in collaborating with statutory bodies and mental health service providers, the level of preferred strategic alliances reflected in the SAF (cooperation, coordination, and collaboration), and any relevant facilitators or barriers (see Additional file 1: Appendix 2. Semi-Structured Interview Guide). For example, the *purpose* of entering and maintaining alliances was explored through questions about the goals and perceived benefits of working with relevant bodies in delivering work-related mental health programmes. *Leadership and decision-making* were explored through questions about power dynamics that would facilitate or challenge the alliance. *Strategies and tasks* were explored through questions about the structures and processes that would support collaboration. Factors potentially influencing c*ommunication and interpersonal relations* were explored through questions about the characteristics of relationships that would support or inhibit the development and maintenance of such alliances. Questions also explored COs’ views about and how such an alliance might work, and potential levels of strategic alliances that these organisations would be willing to engage in, and the reasons this was the case. Probing questions were used when needed to clarify the responses, gain further insights, and overcome researcher and respondent bias [38].

### Coding and data analysis

All interview responses were transcribed verbatim by the first author, then confirmed for accuracy by the second author and imported into NVivo 12 software [39]. Each transcript was de-identified and assigned a unique code relating to the organisation category (ICNPO [36]) and whether they provided a submission to an inquiry (see Table 1). Categorisation and grouping were done to identify any potential differences between types of COs and those that made submissions and those that did not, particularly the interest/motivations, capacity, and facilitators and barriers, in relation to working with relevant bodies in the mental health space. Transcripts were reviewed systematically by the research team’s academic experts with extensive experience in the design, implementation and management of safety, wellbeing and return-to-work programmes following injury, who provided qualitative methods expertise on data analysis and data interpretation.

**Table 1:** Characteristics of Community Organisations (n = 22)

				Contributor, n (%)	Non-contributor, n (%)
**Category**					
		*Culture & Recreation*			
			Sporting clubs	1 (9.1)	3 (27.3)
			Social/service clubs	1 (9.1)	2 (18.2)
			Community radio	1 (9.1)	1 (9.1)
		*Social Services*			
			Family/social services organisations	4 (36.4)	2 (18.2)
			Self-help/support groups	0 (0)	1 (9.1)
		*Development & Housing*			
			Community houses/hubs	3 (27.3)	2 (18.2)
			Employment & training organisations	1 (9.1)	0 (0)
**Location**					
		Metro/urban		7 (63.6)	8 (72.7)
		Regional		4 (36.4)	3 (27.3)

Contributor – CO that provided submissions to mental health reviews; Non-contributor – CO that did not provide submissions to mental health reviews

A combination of inductive and deductive approaches based on Braun and Clarke’s thematic analysis guidelines [40] was used to extract key themes and subthemes guided by the aim of the research and the research questions. The SAF [23] was used to label themes and subthemes specific to the facilitators and barriers influencing COs’ decisions to collaborate with third parties. A flexible and iterative process was used, by which themes and subthemes were identified and continuously adapted and refined as understanding developed [41]. The first author coded responses of a subset of interview transcripts (n = 5) using the SAF framework. The initial codes were checked for emerging patterns and grouped into a draft framework of themes and subthemes. The research team met weekly to discuss, revise, and refine the emerging themes to ensure they are representative of the data [42]. Any differences of opinion were discussed until consensus was reached among the research team. The framework was then applied to the remaining transcripts, whilst allowing for emergent themes until no new themes could be determined [43]. Field notes that were taken during each interview were subsequently used in data analysis discussions among the research team to overcome any potential biases [38]. As an accepted method of ensuring the trustworthiness of qualitative research [44], we calculated the inter-rater reliability using a subset of codes from across a random selection of transcripts [43], which reached 90% agreement.

## Results

### Demographic and sample characteristics

The organisations demonstrating the characteristics identified in previous research [20] (n = 42) were first categorised according to the ICNPO classification system [36], which included Culture & Recreation (CR) (n = 14), Social Services (SS) (n = 18) and Development & Housing (DH) (n = 10). They were then allocated into the ‘Contributor’ (C) or ‘Non-contributor’ (NC) group depending on whether they made a submission to a mental health inquiry and invited to participate in the study. These categories and groups will be used to explore differences in COs’ responses in relation to the aims of this study.

Twenty-two out of forty-two organisations (52.3% response rate) agreed to participate. Half of the COs (n = 11) provided submissions to the mental health reviews (the ‘Contributor’ group), and the other half did not make submissions (the ‘Non-contributor’ group). These groupings will be used to explore differences in COs’ responses regarding approaches that would facilitate their collaboration with third parties to address work-related mental health. There were 13 females and 9 males with a mean age of 44.7 years and a mean tenure of 6 years. Half of the respondents (n = 11) were Chief Executive Officers (CEOs)/Presidents, and the other half were Senior Managers aged between 29 and 66 years.

### Contributors versus non-contributors

First, we explored the reasons why COs made, or did not make, submissions to the mental health reviews (Cs versus NCs). Understanding the reasons is important as it can help identify opportunities to encourage NCs to change perceptions about their potential role in addressing the work-related mental health concerns of their communities and reduce barriers through capacity building and resource provision to empower these organisations to become co-collaborators in work-related mental health initiatives. Those that were NCs, while still interested in addressing the mental health concerns of those they serve, did not make submissions due to three primary reasons; a perceived lack of expertise (*“we’re just not specialised in these issues”*), limitations in their capacity to deliver programmes (*“this would require significant funding and resources that we simply do not have”*), and thinking such activity was out of the scope of their mission (“*we’d have to look at how this would align with our purpose, as this isn’t obvious”*).

**Fig. 1:**
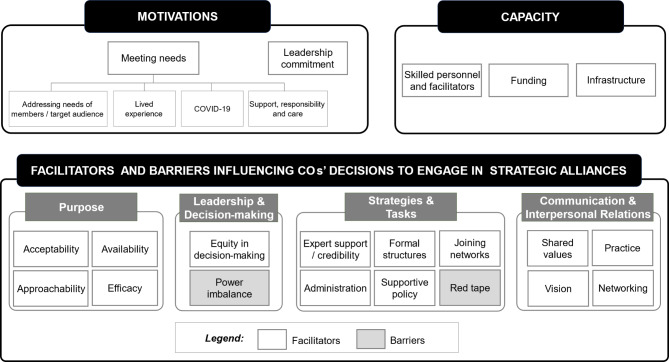
Themes representing the factors influencing community organisations’ decisions to engage in strategic alliances

The following section presents the results of interviews with Cs and NCs in response to each research question. The results associated with RQ1 are presented in two parts including the *categories* of COs considered suitable for delivering mental health programmes by group (Table 2) and a summary of their *motivations* to address work-related mental health and provide relevant mental health information, education and peer support programmes (Table 1). Next, we present results related to RQ2 regarding limitations in COs’ *capacity* to deliver these programmes (Table 3). Finally, we present the results associated with RQ3 which explored the *facilitators and barriers influencing COs’ decisions* to work with relevant bodies to tailor/deliver programmes (Table 4). A visual representation is presented in Fig. 1. It is important to note that the aim of this study was to identify any facilitators or barriers to deliver work-related mental health initiatives, not if there were any characteristics that made organisations more suitable to do so than others.

**Table 2:** Themes for community organisations’ motivations to address work-related mental health

	Contributor (n = 11)	Non-contributor (n = 11)
Meeting Needs (n = 18)	Addressing Needs of Members/Target Audience *“We’d want to make sure that those specific needs…are taken into account.” (SS5,C)*	*“We’d have to look at whether it…is aligned with our organisational priority.” (DH3,NC)*
	Lived Experience *“Lived experience…is critical if these programmes are to be fit for purpose.” (SS6,C)*	*“These programmes need to offer something people can resonate with, by people who’ve been in those situations.” (CR5,NC)*
	COVID-19 *“With COVID-19, we’ve seen an uplift in staff experiencing mental ill-health…but also…increased awareness in…these issues.” (CR4,C)*	*“During COVID-19, we expanded the scope of our services to address mental health…to manage the safety of our communities.” (CR8,NC)*
	Support, Responsibility, and Care *“Our staff feel a strong responsibility to communities to address all sorts of mental health problems.” (CR1,C)*	*“We are not specialised in mental health, but we care for our community…and feel that we have a responsibility…to provide support.” (CR8,NC)*
Leadership commitment (n = 3)	*n/a*	*“Without leadership invested…nothing is going to be successful especially as work-related mental health is not within our scope.” (CR5,NC)*

CR – Culture & Recreation category, SS – Social Services category, DH – Development & Housing category

C – Contributor to mental health reviews, NC – Non-contributor to mental health reviews

**Table 3:** Themes for community organisations’ capacity to deliver work-related mental health information, education and peer support

	Contributor (n = 11)	Non-contributor (n = 11)
Skilled Personnel and Facilitators (n = 14)	*“We don’t really have…expertise in work-related mental health…we would need staff upskilling.” (SS5,C)*	*“We don’t have skilled staff…we’d need to…hire people.” (DH3,NC)*
Funding (n = 10)	*“Without external funding, it would be difficult to engage in this.” (DH5,C)*	*“We would need funding…from government.” (CR6,NC)*
Infrastructure (Online Delivery) (n = 2)	*“We must be mindful whether we have the technical expertise to deliver…online.” (CR9,C)*	*“We need to be aware of any IT requirements in delivering these initiatives.” (CR2,NC)*

CR – Culture & Recreation category, SS – Social Services category, DH – Development & Housing category

C – Contributor to mental health reviews, NC – Non-contributor to mental health reviews

**Table 4:** Themes for the facilitators and barriers influencing Community Organisations’ decisions to engage in strategic alliances

SAF Domain		Theme/Factor	Contributor (n = 11)	Non-contributor (n = 11)
Purpose				
	Facilitators			
		Acceptability (n = 5)	*“The support networks available…don’t necessarily fit our communities’ needs.” (CR1,C)*	*“One of our strengths is determining what the gaps and needs are…and what would work for them.” (SS2,NC)*
		Approachability (n = 5)	*“Something that…people could relate to…would get people in.” (CR4,C)*	*“Many…don’t go to mental health services as they are afraid they might be stigmatised, but feel safe with us.” (DH4,NC)*
		Availability (n = 5)	“*The lack of mental health services is visible in rural areas…That’s where we could help.” (DH5,C)*	*“Working through us is easier to reach more people…than having to work with 60 different organisations.” (DH4,NC)*
		Efficacy (n = 3)	*“We’re…a cost-effective way to benefit people…at risk of mental health issues.” (DH2,C)*	*n/a*
Leadership and decision-making				
	Facilitators			
		Equity in Decision-making (n = 8)	*“…as long as our role and expertise is recognised, they’ll be getting good value from us.” (SS7,C)*	*“That would show us that…what we have to say matters in making decisions.” (CR3,NC)*
	Barriers			
		Power Imbalance (n = 6)	*“There is no point that…white, city people telling remote people what they should be doing, that won’t work.” (SS5,C)*	*“Problems occur if our…professional knowledge is not acknowledged.” (DH3,NC)*
Strategies and tasks				
	Facilitators			
		Expert Support/ Credibility (n = 10)	*“Organisations like occupational health and safety bodies, they have the competence, credibility, and reputation.” (CR4,C)*	*“We’d need to rely on mental health experts to ensure…content fidelity…otherwise it may end up hurting people.” (SS2,NC)*
		Administration (n = 10)	*“Such partnerships would work if…we have formal agreements to help keep us on track.” (DH2,C)*	*“Without central support, things won’t take off or would derail quickly.” (CR5,NC)*
		Formal Structures (n = 6)	*“…part of a mental health alliance…this is how we can add value.” (SS6,C)*	*“An advisory board where we could…develop programmes that would benefit the needs of members.” (DH3,NC)*
		Supportive Policy (n = 3)	*n/a*	*“We’ve got great mental health policies…which fuel public discourse and create a platform for change…” (CR2,NC)*
		Joining Networks (n = 2)	*n/a*	*“Networks…that’s where we make those connections immediately and find ways to collaborate.” (CR7,NC)*
	Barriers			
		Red Tape (n = 2)	*“If you’re mandating us…to take a lead in work-related mental health support, this…won’t be perceived well.” (CR4,C)*	*“We’re not in the business of bureaucracy or reporting.” (CR8,NC)*
Communication and interpersonal relations				
	Facilitators			
		Shared Values (n = 7)	*“There must be really strong common values that organisations share for a partnership to work.” (SS7,C)*	*“It’s that ability to…share the same values.” (CR5,NC)*
		Vision (n = 6)	*“We should have a common…understanding of the outcomes.” (DH6,C)*	*“Organisations…that had a serious commitment…and a long-term view of where the partnership is going.” (CR5,NC)*
		Practice (n = 3)	*“We get to see…how they operate and whether there are opportunities to work together.” (CR9,C)*	*n/a*
		Networking (n = 2)	*“Through networking, we get to see what other organisations are doing.” (CR9,C)*	*“Networking is something that we’re very good at, it’s how we best function.” (CR3,NC)*

CR – Culture & Recreation category, SS – Social Services category, DH – Development & Housing category

C – Contributor to mental health reviews, NC – Non-contributor to mental health reviews

### RQ1 What categories of COs are suited to deliver work-related mental health information, education, and peer support programmes, and what would motivate them to do so?

#### Categories

Nine out of the participating COs were from the CR category, 7 from the SS category, and 6 from the DH category (response rates: 64.3%, 38.9%, and 60% respectively). The profile of COs that participated in this study is presented in Table 2.

#### Motivations

*Meeting Needs.* The strongest, and most consistent overarching theme was meeting the needs of members/target audience (n = 18). Respondents from all CO categories (CR, n = 8; SS, n = 5; DH, n = 5) believed that their involvement; “*comes down to addressing our members’ needs”*. Both groups (C, n = 10; NC, n = 8) demonstrated an awareness of the work contexts that impact the working lives of their members, *“things like bullying, sexist cultures, ineffective policies”*. Broader socio-economic considerations such as *“women’s over-representation in precarious work*”, and the social inclusion of multicultural groups were also discussed by respondents from organisations located in both metro/urban and regional locations (n = 11 and n = 7 respectively). Succinctly, this theme is about *“making sure that anything we do it’s in line with our mission”.* Three more specific motivators were identified as an extension of meeting needs.

*Lived Experience.* COs believed that work-related mental health issues are best addressed by those with a lived experience (n = 8). Respondents (CR, n = 2; SS, n = 3; DH, n = 3) thought that the lived experience of work-related mental injury of their staff/members would enable them to communicate “*what’s going to work and why*”. Both groups (C, n = 5; NC, n = 3) believed that such an approach had the potential to overcome workers’ reluctance to engage.

*COVID-19.* Another subtheme identified was the impact of COVID-19 (n = 6). Respondents (CR, n = 2; SS, n = 2; DH, n = 2) understood this motivator both in terms of increased awareness and impact of mental health issues affecting their members/audience and the community more broadly; “*we see a lot of people who are suffering from mental ill-health as an effect of COVID-19”.* Both groups (C, n = 3; NC, n = 3) reported a willingness to provide support and ensure that *“nobody was left out”.*

*Support, Responsibility, and Care* was the third subtheme identified (n = 4). Cs felt *“a strong responsibility to address these issues”.* In turn, despite not having expertise in work-related mental ill-health, NCs believed they could still help by offering peer support and providing avenues where workers could *“find mates to share their work stresses…without worrying they might be stigmatised”.* Only respondents from the CR category (C, n = 2; NC, n = 2) identified this motivator.

*Leadership Commitment*. While leadership is generally a facilitator, in this case it was identified as a secondary motivator to ensure the success of initiatives addressing work-related mental health by NCs, particularly as these activities were seen as being out of scope (n = 3), *“you need leadership that is committed…or things won’t happen”.* Only respondents from the CR category from metro/urban locations identified this theme.

Table 1 presents the themes relating to COs’ motivations to address work-related mental health arising from interviews.

### RQ2 What is the capacity of these organisations to deliver work-related mental health information, education, and peer support programmes?

*Skilled Personnel and Facilitators.* The main factor identified as influencing COs’ capacity to deliver work-related mental health literacy and peer support relates to having people with the right skills and knowledge (n = 14). Unskilled personnel was acknowledged as a key barrier; *“our staff want to ‘fix’ people, but they’re not…trained to do that”.* Respondents (CR, n = 5; SS, n = 5; DH, n = 4) believed that these initiatives should only be delivered by *“trained facilitators”*. Both groups (C, n = 8; NC, n = 6) saw train-the-trainer programmes such as Mental Health First Aid as critical in building the mental health literacy of frontline staff to *“identify those that suffer from work-related mental ill-health”, “provide peer support”*, and *“make referrals to specialist services”.*

*Funding.* Another key factor identified relates to funding (n = 10). Respondents (CR, n = 4; SS, n = 4; DH, n = 2) believed there was *“scope to expand our services to provide work-related information”*, however, funding restrictions were seen as a critical challenge to delivering *“any services that are not part of our core business”.* Respondents that identified this theme (C, n = 4; NC, n = 6) believed that delivery of these programmes should be subsidised by the government and had limited knowledge of other funding sources/providers.

*Infrastructure (Online Delivery)*. The last factor identified as impacting COs’ capacity is related to the infrastructure required to support online programme delivery (n = 2). The lack of having *“appropriate channels”* and *“the right platforms”* was perceived as potentially a critical barrier. This result is unsurprising, considering that the interviews were conducted during COVID-19 lockdown during which many COs struggled to deliver activities virtually. Only respondents from COs in the CR category (C, n = 1; NC, n = 1) identified this theme.

Examples of feedback for each theme regarding COs’ capacity to deliver work-related mental health programmes are contained in Table 3.

### RQ3 What are the facilitators and barriers influencing COs’ decisions to work with relevant statutory bodies, and mental health service providers to tailor, and deliver work-related mental health information, education, and peer support programmes?

The facilitators and barriers influencing COs’ decisions to work with relevant statutory bodies, and mental health service providers to tailor, and deliver work-related mental health information, education, and peer support programmes (RQ3) are grouped under the four domains of the SAF [23] illustrated in Table 4 and reported below.

### Purpose

#### Facilitators

*Acceptability.* A key purpose identified as influencing COs’ decisions to collaborate relates to improving the acceptability of work-related mental health programmes (n = 5). Respondents (CR, n = 1; SS, n = 3; DH, n = 1) stated that *“one-size-fits-all approaches won’t bring people in”* and believed that their deep understanding of their *“members’ needs and wants”* places them in a unique position to help tailor messages that would improve the acceptability of these programmes for their audiences, *“we can… put things into context and a language that reflects our members’ needs”.* This theme was reported by respondents from both groups (C, n = 3; NC, n = 2).

*Approachability.* Enhancing the approachability of programmes was another key purpose identified (n = 5). Respondents (CR, n = 2; SS, n = 1; DH, n = 2) believed that their organisations’ safe, inclusive, and non-stigmatising environments and lived experience of their members could offer something that *“people can resonate with”*, in a way that *“makes sense for the people who will be benefiting from that support”.* CO leaders from both groups (C, n = 3; NC, n = 2) identified this theme.

*Availability.* The next purpose identified relates to improving the availability of programmes (n = 5). This theme was reported only by respondents from COs in the CR (n = 2) and DH (n = 3) categories. CO leaders (C, n = 3; NC, n = 2) asserted that their organisation could potentially *“fill a gap”* in service provision and provide access to information and support for vulnerable workers such as those with a multicultural background, or living in remote areas; *“they may not be able to see a specialist, but they will come to us”.* Respondents also believed that their organisation’s ability to improve workers’ access was linked to their membership size, *“our reach is enormous, we have 14,000 members”* and trust in the community, *“people don’t have any doubt as to what we stand for…they listen to us”.*

*Efficacy.* The last theme identified in this domain relates to improving the efficacy of programmes (n = 3). Their understanding of members’ needs was seen as helpful in tailoring initiatives in a way that would lead to improved work-related mental health outcomes, as *“if these are not addressed, then no…programme is going to be effective.”* Only respondents from organisations in the DH (n = 2) and SS (n = 1) categories that contributed to mental health inquiries reported this theme.

### Leadership and decision-making

#### Facilitators

*Equity in Decision-making.* The main facilitator identified in this domain relates to COs’ involvement in making decisions (n = 8) by CO leaders from all categories (CR, n = 4; SS, n = 2; DH, n = 2) and groups (C, n = 3; NC, n = 5). Respondents believed that formal recognition of their role/contribution would positively influence interorganisational relationships; *“that shows… we are important to them, that values what we bring…”.* They also reported a preference for equitable relationships where all parties *“get a seat at the table”* and *“have a say in the decisions being made”.*

#### Barriers

*Power Imbalance.* Power dynamics between partners strongly influence COs’ decisions to engage, a perceived imbalance acting as a critical barrier to collaboration (n = 6). This theme was understood by respondents from all categories (CR, n = 2; SS, n = 2; DH, n = 2) and groups (C, n = 3; NC, n = 3) in terms of their organisation’s lack of control over decisions, and inability to influence the terms of engagement. They also believed that power-related challenges could arise if partners were *“too prescriptive about how things should be done”*, or did not take into consideration *“what we had to say…or how we did things”.* In short, if COs were *“brought in at the very end of the process, as an afterthought, and asked to support something that we know it isn’t going to work”.*

### Strategies and tasks

#### Facilitators

*Expert Support/Credibility.* Recommendations and advice from “*those that have the experience, credibility, and expertise in the psychological safety space”* including statutory bodies/regulators and mental health service providers were seen as a key strategy in supporting COs’ involvement (n = 10). Respondents (CR, n = 6; SS, n = 2; DH, n = 2) believed that experts could direct COs to appropriate programmes, as *“there’s a lot out there, and we don’t really know…if their content is evidence-based, or if they have links with organisations that can support people”.* Such guidance was seen as important by respondents from both groups (C, n = 4, NC, n = 6) in overcoming their fear to deliver inadequate content, which “*may do more harm than good”*.

*Administration.* COs’ participation in strategic alliances is highly dependent on having appropriate administrative support (n = 10). Respondents (CR, n = 2; SS, n = 5; DH, n = 3) referred to the benefit of having agreements to *“clarify expectations”.* Delegation of roles was seen as critical in holding *“all parties accountable”.* CO leaders (C, n = 5, NC, n = 5) also believed that the partnership itself required dedicated resourcing to “*develop and nurture the relationship”* as *“it won’t happen by itself”.*

*Formal Structures*. The success of strategic alliances is also dependent on having an appropriate structure in place (n = 6). Respondents from COs within the SS (n = 3) and DH (n = 3) categories (C, n = 4, NC, n = 2) believed that formal structures, such as *“committees”, “advisory boards”*, and *“alliances”* would best support their involvement, leverage their strengths, and utilise their knowledge of members’ needs.

*Supportive policy.* A supportive policy context was the next facilitating factor reported (n = 3). CO leaders highlighted the benefit of policies that promote “*behaviours that contribute to good and bad mental health in the workplace”* in the community to build *“the momentum for action”.* This theme was reported only by respondents within the CR (n = 2) and DH (n = 1) categories that were NCs to mental health reviews.

*Joining Networks.* Notwithstanding the importance of formal structures, having the opportunity to connect with other organisations through joining networks such as *“round tables”*, and *“communities of practice”*, which are based on learning/sharing information for mutual benefit was also identified as an important facilitator (n = 2), *“connectivity…it’s what makes it work for us”.* Only respondents from the CR category that were NCs reported this theme.

#### Barriers

*Red tape.* The key barrier in this domain relates to bureaucratic processes (n = 2). Respondents from the CR category that were NCs that identified this theme described their fear of being pressured to *“do something that is against their DNA”* and *“losing their identity to conform to the rules of a regulated environment”.* They believed that branching into work-related mental health may put them at risk of being “*overwhelmed by bureaucracy”.*

### Communication and interpersonal relations

#### Facilitators

*Shared values.* CO leaders believed that the development and maintenance of productive working relationships are strongly dependent on partners having similar values (n = 7), *“the important thing is that…our values are aligned”*. Respondents from all categories (CR, n = 4; SS, n = 2; DH, n = 1) and groups (C, n = 3; NC, n = 4) described his theme in terms of *“like-mindedness”*, and having a *“deep understanding of each other”.*

*Vision*. All parties sharing goals and having a long-term view were seen as positively influencing the success of an alliance (n = 6). Respondents from all categories (CR, n = 3; SS, n = 2; DH, n = 1) and groups (C, n = 3; NC, n = 3) understood this theme in terms of *“commitment”* and *“agreement on outcomes”.* Simply stated, “*These partnerships bring together people wanting to connect with organisations that have similar goals in mind”.*

*Practice.* The next theme reported relates to practice (n = 3). COs (CR, n = 1; SS, n = 1; DH, n = 1) highlighted the importance of finding *“common ways of operating”* and having “*the same principles of engaging with people”.* This theme was reported only by respondents that were Cs to inquiries.

*Networking.* An important step in developing productive interorganisational relationships involves networking (n = 2). CO leaders that identified this theme saw networking as an opportunity to *“get to know people”, “learn from others”*, and identify common interests or areas for potential collaboration. This theme was only reported by respondents from the CR category that were NCs to mental health reviews.

## Discussion

This is the first research that has used a theory-based decision-making model (the SAF) to explore the motivations and capacity, and the facilitators and barriers for COs to consider delivering work-related mental health initiatives through strategic alliances with statutory bodies and mental health service providers. The results and implications for policy and practice are discussed in relation to the CO groups (Cs versus NCs to mental health inquiries) and categories (CR, SS, DH).

### Group differences

The mental health inquiries were established to determine the interests of organisations in working with relevant bodies and investing resources to improve mental health and wellbeing outcomes within the community but did not explore their interest in extending efforts to address work-related mental health. This study addressed this gap and explored the interest of these organisations in collaborating with statutory bodies and mental health service providers to specifically deliver work-related mental health literacy and peer support. These findings were compared to the group of participant COs that did not make submissions to mental health inquiries (NCs) to explore differences between the two participant groups of COs to identify the reasons for non-submission, not to explore their suitability to address work-related mental health as this had already been determined through the selection process (i.e., appealing to workers, safe environments, serving working aged groups of people).

NCs were reticent to make submissions and reluctant to engage with future work-related initiatives due to perceived limitations in expertise, capacity, and perceived misalignment with their mission. The interview data also indicated that the work-related mental health of their communities was a concern that they were interested in addressing under the right conditions such as resources, support, and expertise building. This suggests that these COs may need to be approached in a manner that addresses perceived barriers to encourage third-party collaboration and persuade them to consider the work-related mental health of their communities as a priority. Aside from reservations about their limitations, NCs identified the same range of facilitators and barriers to successful collaboration as Cs.

### Categories and motivations (RQ1)

#### Categories

This study identified the categories of COs suited to deliver work-related mental health literacy and peer support programmes on a prima facie basis using previous research [20] and the ICNPO classification [36] (CR, SS, and DH). Determining any differences between categories regarding their suitability was outside the scope of this study.

#### Motivations

The overwhelmingly consistent motivation from all COs was their mandate to meet their community needs including mental health. An unexpected finding was the influence of COVID-19 on COs’ increased awareness and impact of mental health issues in the community. While COVID-19 has brought work-related mental health to the forefront of both groups of organisations, a challenge will be to keep their interest alive in the aftermath of this pandemic. Appeals to develop alliances with all COs could be built around increasing awareness of the current and emerging work-related mental health needs of their communities. This may require tailored approaches (peer support, literacy, referral) addressing vulnerable groups of workers such as multicultural, rural/remote, and young workers.

### Capacity (RQ2)

Our study identified the factors that limit the capacity of COs from all groups and categories to deliver work-related mental health programmes, including a lack of skilled personnel/facilitators, funding and infrastructure constraints. As COs rely heavily on untrained volunteerism [17], initiatives such as ‘train-the-trainer’ could equip frontline CO staff to recognise the signs of work-related mental injury, have safe conversations, and provide referrals to specialised support [45]. Our results support previous literature findings, which show that a precarious and highly competitive funding environment impact COs’ capacity to secure and maintain funds necessary to support programmatic delivery [46]. COs’ funding limitations and reliance on government grants/sponsorships could potentially be overcome by raising their awareness of alternative funding opportunities such as philanthropic and corporate grants [47]. A framework directing the development of alliances needs to consider the scale and types of initiatives, however, must be adapted to meet the infrastructure limitations of COs, particularly those from the CR category.

### Facilitators and barriers (RQ3)

The SAF’s domains were used also to identify COs’ perceived facilitators and barriers to the establishment and maintenance of strategic alliances.

*Purpose.* The key purpose identified by our study as facilitating engagement of COs from all groups and categories in strategic alliances with relevant bodies resides in improving the acceptability, approachability, availability, and efficacy of work-related mental health programmes. COs’ views of their unique strengths, comprising socially-inclusive environments, reach, and relatability of members with lived experience confirm the attributes identified by a previous study as the very reason why workers believed that these organisations would be suitable in addressing their mental health needs [20]. Literature shows that the ‘fit’ between the perceived characteristics of service providers and the needs of individuals is critical in improving access to mental health services [48, 49].

*Leadership and decision-making.* COs’ decisions to enter, and maintain engagement, within these alliances are influenced by their perceptions of leadership and decision-making. Our findings support previous literature, showing that COs’ contributions in cross-sector partnerships are generally undervalued due to a lack of understanding of their strengths [50, 51]. In addition, COs lack the decision-making authority compared to government or corporate partners [24]. COs’ concerns regarding power imbalance could be overcome by promoting equitable relationships, where all parties have a say in decision-making, and control over the terms of engagement [27, 52].

*Strategies and tasks.* Our study identified several strategies that could support COs’ decisions to engage and enhance their co-ownership of work-related mental health outcomes. First, as COs do not have work-related mental health expertise, guidance from statutory bodies and mental health organisations could improve their awareness of effective programmes and subsequently their willingness to promote these initiatives more broadly within their communities.

Second, coordination of activities through a central entity such as a community peak body could alleviate some of the COs’ capacity limitations and administration concerns [21, 51].

Third, having a supportive structure that is perceived as appropriate is also critical to COs’ engagement. Policymakers and practitioners should be mindful of the types of structures that could best support COs’ involvement based on their motivations, capacity, and category, and provide them with a range of opportunities to participate. While we expected COs to want to be included in formal structures such as advisory/working groups, results show that networks are also important in paving pathways for their engagement. Cooperative approaches such as joining networks with like-minded parties may help COs, particularly those from the NC group, to identify relevant agents, and structures that could support their deeper involvement on the strategic alliance continuum (i.e., cooperation and coordination through to collaboration).

Fourth, policymakers should be aware of the impact of the policy context on COs’ decisions to collaborate. OHS campaigns could improve the awareness of mental health issues arising in the workplace of COs that were not involved with reviews, particularly those within the CR and DH categories, which in turn may facilitate their involvement.

Fifth, statutory bodies/regulators should be mindful of COs’ fear of losing their autonomy due to red tape, particularly those from CR and DH categories. COs are often asked to deliver services in prescribed agreements [53]. Stringent public sector commissioning processes have also been shown to compromise the ethos of COs [24, 54]. As COs are often resource and time-poor, their involvement needs to be done in a supportive manner.

*Communication and interpersonal relations.* Shared values and agreement on outcomes (vision) were found to be critical in maintaining positive working relations with COs from all categories and groups. Literature shows that collaborations are most productive when members connect with each other on a personal level, and have similar values and goals [28, 55]. Networking could expose COs to what other organisations were doing, and their ways of operating, and identify collaboration opportunities.

### Implications

Our study employed the SAF to identify the optimal approaches to initiate and enhance productive working relationships focused on addressing work-related mental health with a wide range of COs and include cooperative, coordination and collaborative approaches. The mission/scope of services, size, staffing, level of resources, and audience needs vary significantly between different groups, and categories of COs, which subsequently impact the scale and type of initiatives that these organisations may be able, and willing to provide. Some COs may be better suited to deliver work-related mental health literacy initiatives, while others to provide peer support. However, third-party contribution is still required to guide, and an appropriate framework designed to support them in these endeavours with expertise, resources, and structures/networks in a manner that is empowering not controlling them.

### Limitations and suggestions for future research

As this is an exploratory study, an important limitation is that the small purposive sample size of our study reduces the generalisability of results. Respondents were from large organisations which have more resources, connections, and collaborative experience than most COs, and may introduce a response bias. Further, respondents were predominantly from metro/urban areas and therefore may have different perspectives than those located in rural or remote settings. Another limitation is that the sample did not include COs that are not providing mental health initiatives, although they possessed the attributes that would make them an appealing mental health literacy programme provider. In addition, a comparison of differences between categories of COs and their potential to engage in different levels of strategic alliance was beyond the scope of this study and was also limited by the sample size. Future research could explore a range of a greater number and broader categories of COs in relation to differences in their preferred level of strategic alliance according to the SAF, in addition to the types of programmes most suited to address the specific needs of at-risk groups of workers they serve.

Finally, whilst our study was theory-driven and contributes to current academic literature, a major strength is that it addresses a critical policy issue by utilising theory rather than testing the efficacy of theory itself. The findings can ultimately be used to develop models of cross-sector strategic alliance to address critical public policy issues at the community level. Future studies should build on this research by investigating the willingness of statutory bodies and mental health service providers to initiate and support COs’ efforts in delivering work-related mental health initiatives. A framework to develop cross-sector relationships between key stakeholders need to consider the impact of size, mission/scope, level of staffing/resources of various groups, and categories of COs to identify where these organisations may be placed on the strategic alliance continuum, and the impact of the size, category, mission/scope, and resources of COs in the design of approaches that can meet their specific needs and empower them to expand the reach of programmes addressing worker mental health literacy, and help-seeking behaviours.

## Conclusion

In Australia [1] and internationally [4] there is a pressing need to mobilise efforts across sector organisations to address the escalating rates of mental injury. This research responds to calls arising from recent mental health reviews to utilise community-based resources such as community organisations to address work-related mental ill-health. Guided by collaboration theory, this study used a qualitative approach to explore the motivations influencing COs’ potential willingness to engage with statutory bodies and mental health service providers to expand opportunities for workers to access mental health literacy resources and peer support.

The application of a collaboration theory-based decision-making model (the SAF) was critical in guiding the identification of the resources, structures, and processes that could overcome CO leaders’ perceived limitations to collaborate. The purpose of strategic alliances with COs should be based on what these organisations see as their core attributes and reasons for existing. Our research found that COs that did not contribute to mental health reviews believed that limitations in their expertise, capacity, and scope of activities negatively impacted their efficacy to support mental health initiatives beyond their current efforts. Strategies addressing these perceived limitations through expertise, resources, and administrative support provided by appropriate third parties may improve their confidence and willingness to engage. Promoting equitable relationships could overcome COs’ concerns regarding lack of control over decision-making. Our study identified several strategies that could activate COs’ co-ownership of work-related mental health outcomes, including expert guidance, a supportive policy context, and a range of formal and informal structures. An understanding of a CO’s need to maintain their organisation’s identity is, however, important to maintain its engagement. Administrative support could overcome these organisations’ concerns regarding red tape. Focus should be placed in the early stages of developing alliances, to reconcile any differences in values, norms, and goals. We also found that the motivations, capacity and nature of different categories of COs that participated in the study could potentially inform the scale and types of initiatives they are willing/able to provide (i.e., literacy/peer support), and the level of reliance, on and engagement with, relevant bodies.

In summary, alliances leveraging the infrastructure of statutory bodies/regulators, the expertise of mental health service providers, and COs’ resources, reach in the community, and insights into the needs of the communities they serve could complement existing workplace and public health initiatives by providing workers with greater opportunities to access mental health information and support to prevent, or limit, the escalation of mental injury. This research recommends that a framework guiding strategic alliances between COs, statutory bodies, and mental health service providers should address the type, size, and resources of these organisations and the level of ‘back-room’ support they need if they are to effectively address the pervasive problem of work-related mental injury.

### Electronic supplementary material

Below is the link to the electronic supplementary material.

Supplementary Material 1

## Data Availability

(ADM) The full database of community organisations’ search results retrieved during the study are available from the corresponding author on reasonable request. Given the richness of the qualitative data and the potential for identifying the key informants who participated in the semi-structured interviews, our study full dataset will not be shared publicly but are available from the corresponding author on reasonable request. For any requests or information about the data from this study, please contact Corina Crisan at corina.crisan@monash.edu.

## Abbreviations

CO: Community Organisation
OHS: Occupational Health and Safety
SAF: Strategic Alliance Framework
ICNPO: International Classification of Non-Profit Organisations
CR: Culture & Recreation
SS: Social Services
DH: Development & Housing
