# Supplementary material for: Mobilising strategic alliances with community organisations to address work-related mental injury: a qualitative study guided by collaboration theory
Source: BMC Public Health. 2023 Nov 16;23:2258. doi: 10.1186/s12889-023-17170-w (PMC10652450; doi:10.1186/s12889-023-17170-w)
Supplement: Supplementary file 1 — Supplementary Material 1 [file 12889_2023_17170_MOESM1_ESM.docx]

**Additional file 1: Appendix 1.** Characteristics of Community Organisations identified and recruited. **Appendix 2.** Semi-Structured Interview Guide.

# **Additional file 1.**

## *Appendix 1. Characteristics of Community Organisations identified and recruited.*

|  |  | | | **Contributor** | | | **Non-contributor** | |  |  |  |  |
| --- | --- | --- | --- | --- | --- | --- | --- | --- | --- | --- | --- | --- |
|  | | | | Identified | Recruited | Identified | | Recruited |  |  |  |  |
| **Category** | | | |  |  |  | |  |  |  |  |  |
|  |  | Culture&Recreation | |  |  |  | |  |  |  |  |  |
|  |  |  | Sporting clubs | 2 | 1 | 4 | | 3 |  |  |  |  |
|  |  |  | Social and service clubs | 1 | 1 | 4 | | 2 |  |  |  |  |
|  |  |  | Community radio | 2 | 1 | 1 | | 1 |  |  |  |  |
|  |  | Social Services | |  |  |  | |  |  | | |  |
|  |  |  | Family/social services organisations | 10 | 4 | 2 | | 2 |  |  |  |  |
|  |  |  | Self-help/support groups | 2 | 0 | 4 | | 1 |  |  |  |  |
|  |  | Development&Housing | |  |  |  | |  |  | | |  |
|  |  |  | Community houses/hubs | 3 | 3 | 4 | | 2 |  |  |  |  |
|  |  |  | Employment&training organisations | 2 | 1 | 1 | | 0 |  |  |  |  |
| **Location** | | | |  |  |  | |  |  |  |  | |
|  |  | Metro/urban | | 14 | 7 | 11 | | 8 |  | | |  |
|  |  | Regional | | 8 | 4 | 9 | | 3 |  | | |  |
|  |  | Total | | 22 | 11 | 20 | | 11 |  | | |  |
| Contributor – CO that provided submissions to mental health reviews  Non-contributor – CO that did not provide submissions to mental health reviews | | | | | | | | |  |  |  |  |

## *Appendix 2. Semi-Structured Interview Guide.*

**Introduction**

- Provide a brief overview of the purpose of the study
- Go through the Explanatory Statement and ask if participant/s have any questions
- Explain work-related mental injuries
- Provide examples of mental health literacy programmes addressing mental health issues (including those work-related)
- Collect Consent Form/s

**Reasons why they made submissions, or not, to the mental health reviews**

1. Can you tell me more about why you (your organisation) made / did not make a submission to these reviews?
2. What sort of mental health information/programmes does your organisation provide?
   - Why? Is there a specific need you are trying to address?
3. What can you tell me about the mental health needs of your members/users? Have you noticed a trend?

**Appetite to address work-related mental injury**

1. To what extent do you believe that the mental health issues of members/users may have been caused by work?
   - How do you know that?
2. Would you be interested in providing specific work-related mental health initiatives?
   - Why? Which ones? (explore)

**Capacity to deliver work-related mental health literacy and peer support programmes**

1. What can you tell me about your organisation’s capacity/role to deliver such programmes? (explore resources, personnel, size)
2. What would make it easier for you to deliver them? (explore structures, processes)
3. Is there anything that would prevent you from delivering them?

**Facilitators/barriers to developing/maintaining strategic alliances with vested bodies**

1. What is your experience in collaborating with statutory bodies/mental health service providers?
   - Can you think of an example that worked, and why?
   - How about an example that didn’t work, and why?
2. Would you be interested in working with these bodies to tailor/deliver work-related mental health literacy and peer support programmes?
   - Purpose (goals/perceived benefits of working with these bodies in delivering such programmes)
   - Leadership/decision-making (power dynamics that would facilitate/challenge the alliance)
   - Strategies and tasks (structures/processes that would support/challenge the alliance)
   - Communication/interpersonal relations (characteristics of relationships that would support/inhibit the development of alliances)
3. How do you think such an alliance might work? How? Why? (explore potential levels of integration, i.e., cooperation, coordination, collaboration)
4. What would positively influence your engagement with these organisations?
5. What would stop you from collaborating with them?
